# Intrusive Mental Imagery in Psychological Disorders: Is the Self the Key to Understanding Maintenance?

**DOI:** 10.3389/fpsyt.2015.00103

**Published:** 2015-07-17

**Authors:** Soljana Çili, Lusia Stopa

**Affiliations:** ^1^London College of Fashion, University of the Arts London, London, UK; ^2^Psychology Academic Unit, University of Southampton, Southampton, UK

**Keywords:** mental imagery, intrusions, psychological disorders, trauma, self-defining memories, self

## Introduction

Intrusive mental imagery is a transdiagnostic process (1) present in many psychological disorders including trauma-related disorders; anxiety, mood, and eating disorders; as well as severe mental health problems, such as bipolar disorder and psychosis. In this article, we summarize some of the current literature on intrusive images and then argue that one critical way in which they maintain disorders is through their representation and relationship with patients’ sense of self. We conclude by briefly discussing the treatment implications of this position.

## Characteristics of Intrusive Images

Intrusive images are vivid and, although the visual elements are predominant, they often include other sensory modalities. Cutaneous sensations (e.g., the clothes being tight) and organic elements (e.g., a sense of heaviness), for example, are characteristic in bulimia nervosa (2). Images experienced by patients with vomit phobia can include physical sensations (e.g., being sick), auditory elements (e.g., others’ disgusted reactions), smells (e.g., bleach), and tastes (e.g., recently consumed food) (3).

Intrusive images are often recurrent [e.g., Ref. (2, 4–6)] and can be triggered by specific stimuli (both situational and internal). Social situations (and their anticipation or recall) cue self-images in social anxiety disorder (4, 7), whereas binge eating and weight/shape concerns may activate images in bulimia nervosa (2, 8). Worry or anxiety about appearance can trigger images for individuals with body dysmorphic concerns (9), and in vomit phobia stomach sensations such as nausea or external stimuli such as seeing another person looking unwell act as cueing situations (3).

Image content varies according to the disorder. Sometimes, there is a close correspondence between imagery and patients’ verbal cognitions. For example, persecutory delusions may be accompanied by persecutory images, such as being put in an oven (10) or pharmacy staff tampering with medication to poison the patient (11). In some cases, images link to catastrophic fears – physical or mental catastrophes in agoraphobia (e.g., passing out while crossing the road) (12, 13), making a fool of oneself in social anxiety disorder (4, 14), being contaminated in obsessive–compulsive disorder [OCD; (15–17)], vomiting in vomit phobia scenarios (3), or having a serious illness and dying in health anxiety (5, 18). In other cases, images relate specifically to the physical body. Patients with body dysmorphic disorder experience exaggerated pictures of and sensations such as tingling in the body parts of concern (6). Patients with bulimia nervosa experience images of the self being overweight and unattractive and sensations such as feeling bloated (2, 8). The correspondences between fears and intrusions, however, are not always so immediate or so closely linked. Individuals with depression and bipolar disorder, for example, often report images related to interpersonal problems, including isolation and victimization (19–22).

The perspective from which patients experience intrusions varies across disorders. Patients with disorders like bulimia nervosa (2), social anxiety disorder (23), and body dysmorphic disorder (6) tend to see themselves from an observer perspective. By comparison, patients with disorders like OCD (15–17) are more likely to experience images from a field perspective. Perspective is potentially important because it may influence the images’ emotional and behavioral impact. The field perspective may be associated with more intense emotions [see Ref. (24, 25)], whereas the observer perspective may exert a greater influence on individuals’ future behavior (26).

## Origin of Intrusive Images

Intrusive images are often related to memories of past adverse experiences. In disorders like post-traumatic stress disorder [PTSD; (27–29)], agoraphobia (12), health anxiety (18), bipolar disorder (30), OCD (17), and psychosis (10), intrusions are frequently associated with traumatic experiences, such as being physically or sexually assaulted/abused. In other cases, intrusions originate from less severe experiences, such as arguing with significant others or being teased, criticized, bullied, and humiliated [e.g., body dysmorphic disorder (6), depression (21), social anxiety disorder (4), agoraphobia (12), bipolar disorder (31), OCD (17), bulimia nervosa (8)]. Memories related to feeling bad about one’s self-image and self-consciousness about one’s appearance are also associated with intrusive images; characteristic examples are body dysmorphic disorder (6), bulimia nervosa (2), and social anxiety disorder (4).

Not all intrusive images arise directly from an adverse experience or correspond exactly to what happened during this experience. Some patients experience images that are partly or entirely products of their imagination. In PTSD, for example, intrusive images may be composites formed from fragments of several events (e.g., repeated traumas) or worst-case scenarios about what could have happened during a traumatic event (32, 33). In depression, images may be flash-forwards to imagined future suicide attempts (34, 35).

## Role of Images in Disorder Onset and Maintenance

Evidence suggests that intrusive images play a role in both the onset and the maintenance of psychological disorders. Many patients suffering from health anxiety (5), agoraphobia (12), body dysmorphic disorder (6), and social anxiety disorder (4) report that their symptoms either appeared or intensified after the intrusion-related adverse experience. When it comes to maintenance, imagery may play a significant role through its impact on emotions. Intrusive images are frequently associated with negative emotions, such as fear, helplessness, anger, guilt, anxiety, shame, and disgust [e.g., Ref. (11, 17, 19, 22)]. Their emotional impact is also associated with specific behavioral responses that themselves contribute to disorder maintenance. In OCD, intrusive images elicit anxiety, which often triggers rituals such as washing or checking (15, 17, 36). In bulimia nervosa, images and negative emotions elicited after a binging episode may lead to self-induced vomiting (8). In bipolar disorder, images may amplify positive and negative emotions, thus contributing to the patients’ unstable mood (37, 38). In health anxiety, images may trigger behaviors such as reassurance seeking and body/health checking (5). In PTSD, they may contribute to a sense of impending threat and the use of maladaptive coping strategies (e.g., avoidance) (39). In social anxiety disorder, images often trigger safety behaviors such as internal monitoring that have a detrimental effect on social performance (7, 40–42). They may also be involved in the “post-mortem” of social interactions that patients with this disorder conduct [see Ref. (43)]. In generalized anxiety disorder (GAD), images may play a rather different role. Persistent verbal worry, which is a characteristic of GAD and helps maintain it, may be an attempt to avoid the emotional impact of imagery (44). This may explain the brevity and low frequency of images reported by patients with GAD (45).

## Relevance of Images for the Self

Intrusive images often represent the self and incorporate meanings about the self that are derived from the original memory (e.g., “*I am unlovable/powerless/a failure*” or “*I deserve to be punished*”) [e.g., Ref. (10, 12, 17, 18)]. We believe that the relationship between images, memory, and the self has not been sufficiently explored in the literature and that its analysis would pay dividends both in understanding how self-images contribute to the maintenance of psychological disorders and how best to target them in treatment. The concept of a self-defining memory is a useful starting point. Self-defining memories are vivid, emotional, highly accessible memories that are related to individuals’ most important concerns and conflicts and contain knowledge about their progress in goal attainment (46, 47). As described earlier, many intrusive images are linked to memories and represent critical meanings about the self. Sometimes, these self-images confirm beliefs about the self (e.g., “*I am foolish*” in social anxiety disorder). At other times, as in the case of PTSD, they may be linked to a self-defining memory which has transformed the self in some important way (e.g., “*I am no longer the person I was*”).

The majority of work on intrusive self-images to date has recognized that there may be associated memories and has highlighted the link to beliefs, emotions, and behavior. However, it has not investigated the role of patients’ broader sense of self in these links and the mechanisms that are set in motion when intrusive self-images are activated and that may produce specific emotional and behavioral responses. Our position is that intrusive self-images can only be fully understood by examining the links between self and memory and the self-memory system (SMS) model (48–51) offers one way to do this. According to the SMS model, self-images and goals are part of the working self, the representation of the self that is active at any one time. Working selves are activated when individuals face a shift in environmental demands that requires adaptive responding. They modulate individuals’ cognitive, affective, and behavioral responses to this shift. The aim of the working self – and that of the SMS in general – is to help individuals adapt to their current circumstances while maintaining a stable, coherent sense of self over time (48).

When a traumatic event occurs, the goals of the active working self may not match the situation and therefore cannot guide its processing (50, 51). Moreover, the achievement of these goals is thwarted and this threatens the stability of the self. Consequently, the trauma is either not encoded or is encoded but not integrated with the individual’s autobiographical knowledge base (50). The trauma thus remains highly accessible and associated with the working self that was active when it occurred [see Ref. (52)]. In an attempt to preserve the coherence of individuals’ long-term sense of self, the working self may attempt to inhibit or distort the intrusive memories (e.g., through maladaptive beliefs). In addition, trauma-related images may become associated with goals which try to avoid the state of the world that these images represent (e.g., panic attack, public humiliation) by increasing the discrepancy between them and individuals’ actual state (48, 49).

We have used the SMS model to investigate the impact of self-defining memories on the self (53). We confirmed the hypothesis that these memories and associated images influence the working self and may produce specific emotional and behavioral responses. Specifically, we found that participants reported different aspects of the self (e.g., goals, emotion-related self-cognitions, state self-esteem) after recalling self-defining memories, depending on the valence of the memory (positive or negative) and the extent to which they had derived meaning from it (53). Based on these findings and on the research evidence reviewed in this article, we have proposed that the intrusive images experienced by patients are part of working selves related to adverse events (53). When patients encounter situations that remind them of these events, they do not simply experience the activation of related images: they experience the activation of an entire working self consisting of the images, negative self-beliefs, and goals that aim to distance them from the failure- or threat-related standard represented by the intrusive image(s). This may explain why patients report negative emotions and engage in maladaptive behaviors when intrusions come to mind. For obsessive–compulsive patients, washing or checking rituals (15, 17) may be a way of coping with the intrusion-related anxiety and of distancing themselves from the feared scenarios represented in the intrusions (e.g., contamination). For bulimic patients, self-induced vomiting (8) may be a way of coping with the anxiety triggered by the images of the overweight self and of ensuring that these images do not become reality.

## Relationship between Intrusions and the Self: Directions for Future Research and Implications for Therapy

Research on the impact of intrusions on the working self is still in its infancy. We believe that this area of research may advance our understanding of the maintaining role of intrusive images in psychological disorders and – crucially – may illuminate the mechanism of change behind therapeutic techniques, such as imagery rescripting, that specifically target self-images and associated meanings. Understandably, to date, clinicians have focused primarily on the symptom outcomes of imagery interventions. We know, for example, that imagery rescripting effectively alleviates symptoms in several disorders, including PTSD (54, 55), acute stress disorder (56), depression (57), and social phobia (58–60). Because this technique helps patients modify the beliefs associated with their intrusions, it is reasonable to assume that it changes their sense of self. To date, this change and its possible relation to patients’ symptomatic relief have not been studied in depth. We recommend that future clinical research relies on cognitive and social psychological evidence and theories on the self to investigate the impact of self-defining memory recall and intrusion activation on patients’ sense of self, as well as the way in which therapeutic interventions modify this impact. We also recommend that clinicians working with patients who experience intrusions explore and focus not only on self-beliefs but also on the whole sense of self. We believe that, by focusing more on the self, we may be able to not only help patients recover but to also reduce the chances of relapse by teaching them to exert greater control over the activation of negative working selves associated with adverse experiences.

## Author Contributions

SÇ reviewed the literature and drafted the manuscript. LS contributed to and edited the manuscript. Both authors gave their approval for the final version of the article to be published.

## Conflict of Interest Statement

The authors declare that the research was conducted in the absence of any commercial or financial relationships that could be construed as a potential conflict of interest.
